# Specific sulfation and glycosylation—a structural combination for the anticoagulation of marine carbohydrates

**DOI:** 10.3389/fcimb.2014.00033

**Published:** 2014-03-06

**Authors:** Vitor H. Pomin, Paulo A. S. Mourão

**Affiliations:** Program of Glycobiology, Institute of Medical Biochemistry Leopoldo de Meis, University Hospital Clementino Fraga Filho, Federal University of Rio de JaneiroRio de Janeiro, Brazil

**Keywords:** algae, carbohydrate-based drug development, fucosylated chondroitin sulfate, sea cucumber, sea urchin, sulfated galactan, sulfated fucan

## Abstract

Based on considered achievements of the last 25 years, specific combinations of sulfation patterns and glycosylation types have been proved to be key structural players for the anticoagulant activity of certain marine glycans. These conclusions were obtained from comparative and systematic analyses on the structure-anticoagulation relationships of chemically well-defined sulfated polysaccharides of marine invertebrates and red algae. These sulfated polysaccharides are known as sulfated fucans (SFs), sulfated galactans (SGs) and glycosaminoglycans (GAGs). The structural combinations necessary for the anticoagulant activities are the 2-sulfation in α-L-SGs, the 2,4-di-sulfation in α-L-fucopyranosyl units found as composing units of certain sea-urchin and sea-cucumber linear SFs, or as branching units of the fucosylated chondroitin sulfate, a unique GAG from sea-cucumbers. Another unique GAG type from marine organisms is the dermatan sulfate isolated from ascidians. The high levels of 4-sulfation at the galactosamine units combined with certain levels of 2-sulfation at the iduronic acid units is the anticoagulant structural requirements of these GAGs. When the backbones of red algal SGs are homogeneous, the anticoagulation is proportionally dependent of their sulfation content. Finally, 4-sulfation was observed to be the structural motif required to enhance the inhibition of thrombin via heparin cofactor-II by invertebrate SFs.

## Introduction

Marine organisms represent a very special source of potential therapeutic molecules with unique structures. Among innumerous of these compounds, the sulfated polysaccharides (SPs) have awakened great interest in the scientific community. This happens mostly because of the fact that these compounds hold the characteristic of being naturally polyanionic. This feature makes the SPs suitable to interact with important functional proteins, especially those involved in the balance of health and disease. The nature and quality of the SP-protein interactions control and regulate the activity of these functional proteins in the body. Although the affinity of these molecular complexes are mostly driven by electrostatic interactions in which sulfation content of the SPs play a crucial role, the overall structural features of the SPs are still more influential in the process (Pomin, 2009). The structural features of the SPs involved into the quality of these molecular interactions are sulfation patterns and glycosylation. This latter includes anomeric and enantiomeric configurations, glycosidic linkage position, monosaccharide type and composition.

In the last 25 years, our group has made great efforts in scientific researches related with marine and medicinal glycobiology. As a consequence, many sulfated fucans (SFs), sulfated galactans (SGs) and glycosaminoglycans (GAGs) of new structures have been characterized and described (Vieira and Mourão, 1988; Mourão, 2004; Pomin and Mourão, 2008). We believe that we have fully characterized over 20 new structures of these marine sulfated polysaccharides (MSPs) in our studies (Vieira and Mourão, 1988; Mourão, 2004; Pomin and Mourão, 2008; Pomin, 2009, 2012a,b, 2014a,b). We have also submitted most of these new MSPs to *in vitro* experiments to assess their possible anticoagulant effects (Mourão, 2004; Pomin and Mourão, 2008; Pomin, 2009). Curiously, we noticed that even though bearing significant levels of sulfation, some of these MSPs have insignificant effects toward the coagulation system; while other MSPs, even carrying lower sulfation content, can show surprising levels of anticoagulant activity (Mourão, 2004; Pomin, 2009). Moreover, some SFs and SGs, even though exhibiting nearly equal sulfation levels, but within different sulfation patterns, have completely different anticoagulant effects. This observation has clearly proved the concept that sulfation and thus electronegative-charge density in marine carbohydrates are not the solely structural determinants for the resultant anticoagulant activities of these molecules.

Since we have characterized and used many different structures in our anticoagulant tests across the last years, we should be able to state by now some of the structural features of the MSPs necessary for their differential anticoagulant properties. Based on comparative and systematic analyses on the structure-anticoagulation relationships of certain MSPs, we noticed that to achieve a good anticoagulant response, certain structural combinations of sulfation and glycosylation are indeed required. In these analyses, we have particularly given more preference to examine SFs, SGs and GAGs from marine invertebrates or red algae, since these organisms can provide molecules of well-defined chemical structures (Mourão, 2004; Pomin, 2009, 2012c; Pomin and Mourão, 2012). This kind of structural regularity has facilitated interpretation and this in turn enables us to establish advanced structure-anticoagulation relationships (Pereira et al., 2002; Pomin, 2009, 2012c). Here, we describe based on data of some of our previous works, the major structural combinations of the invertebrate and red algal MSPs that have been proved to be necessary to make a satisfactory anticoagulant effect.

### The role of SFs and SGs of well-defined structures: algal vs. invertebrate molecules

**Figure 1:**
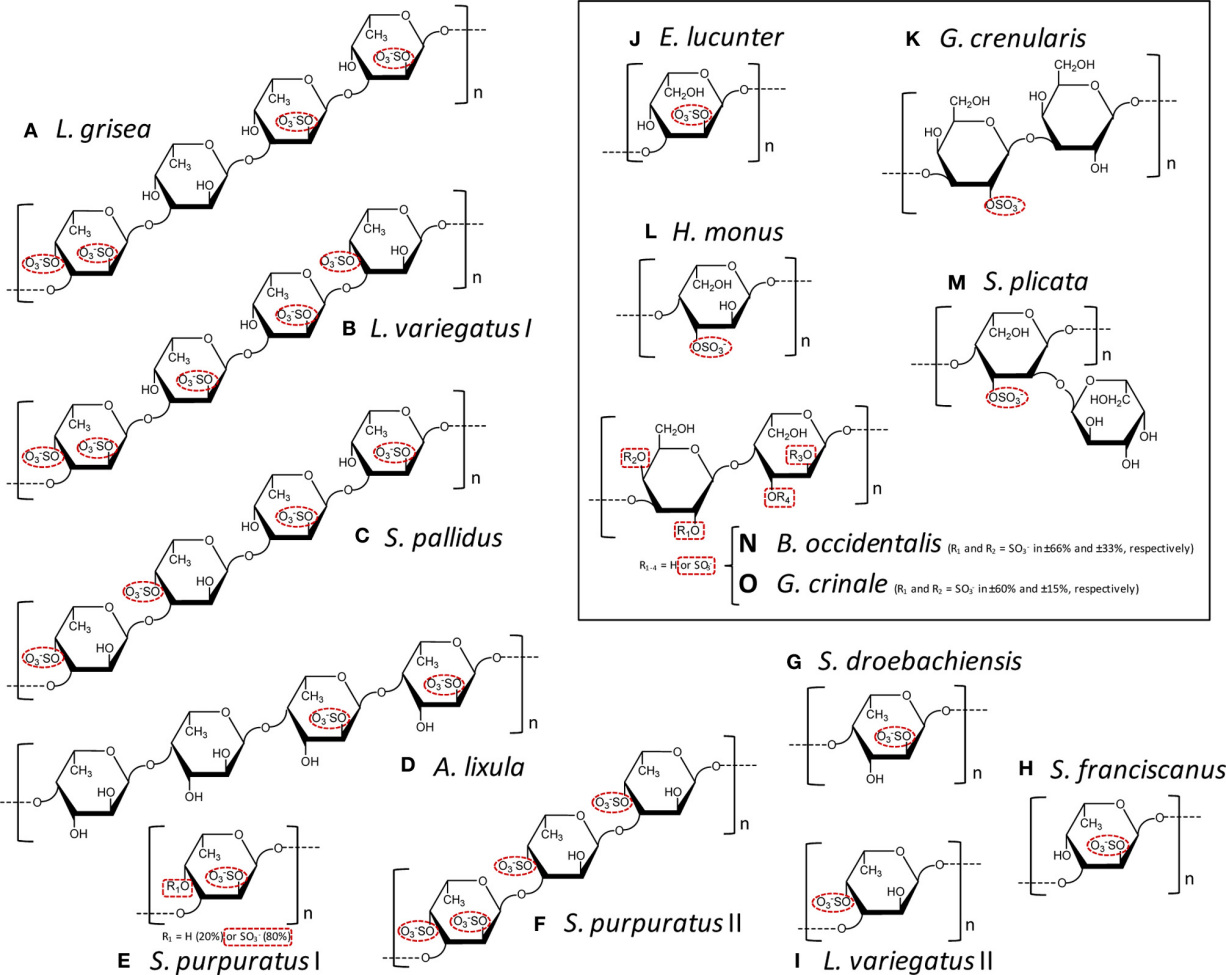
**Structural representation of the repetitive oligomeric units of the SFs (A–I) and SGs (J–O, inside the box) of well-defined chemical structures isolated from cell wall of sea cucumbers (A), from egg jelly coat of sea urchins (B–K), from tunic of ascidians (L,M), and from cell wall of red algae (N,O)**. Modified with permission from Pomin (2012b). These MSP are composed of α-L-fucopyranoses (α-l-Fuc*p*) **(A–I)**, α-L-galactopyranoses (α-L-Gal*p*) (I, M-O), or β-D-galactopyranosyl units (β-D-Gal*p*) **(K,N,O)**. The species-specific structures vary in sulfation patterns (exclusively 2-*O*- and/or 4-*O*-, or 3-*O*-positions), in glycosidic linkages: α(1→3) **(A–C,E,F,H–K)**, α(1→4) **(D,G,L,M)**, β (1→3) **(K)**, or alternating β (1→4) and α(1→3) **(N,O)**; and in number of composing residues of the repetitive units: tetrasaccharides **(A–D)**, trisaccharides **(F)**, disaccharides **(K,N,O)**, or monosaccharides **(E,G–J,L)**, all in linear chains, except **(M)**. The sulfation groups are highlighted by red dashed ellipses or rectangle when just percentage can be estimated. The structures are the following: **(A)** *Ludwgothurea grisea* [→3)-α-L-Fuc*p*-2,4(OSO_3−_)-(1→3)-α-L-Fuc*p*-(1→3)-α-L-Fuc*p*-2(OSO_3−_)-(1→3)-α-L-Fuc*p*-2(OSO_3−_)-(1→]_*n*_ (Mulloy et al., 1994); **(B)** *Lytechinus variegates* I [→3)-α-L-Fuc*p*-2,4(OSO_3−_)-(1→3)-α-L-Fuc*p*-2(OSO_3−_)-(1→3)-α-L-Fuc*p*-2(OSO_3−_)-(1→3)-α-L-Fuc*p*-4(OSO_3−_)-(1→]_*n*_ (Mulloy et al., 1994); **(C)** *Strongylocentrotus pallidus* [→3)-α-L-Fuc*p*-4(OSO_3−_)-(1→3)-α-L-Fuc*p*-4(OSO_3−_)-(1→3)-α-L-Fuc*p-*2(OSO_3−_)-(1→3)-α-L-Fuc*p-*2(OSO_3−_)-(1→]_*n*_ (Vilela-Silva et al., 2002); **(D)** *Arbacia lixula* [→4)-α-L-Fuc*p*-2(OSO_3−_)-(1→4)-α-L-Fuc*p*-2(OSO_3−_)-(1→4)-α-L-Fuc*p*-(1→4)-α-L-Fuc*p*-(1→]_*n*_ (Alves et al., 1997); **(E)** *Strongylocentrotus purpuratus* I ~80% [→3)-α-L-Fuc*p*-2,4(OSO_3−_)-(1→]_*n*_ and ~20% [→3)-α-L-Fuc*p*-2(OSO_3−_)-(1→]_*n*_ (Alves, Mulloy, Moy, Vacquier and Mourão, 1998) and **(F)** *S. purpuratus* II [→3)-α-L-Fuc*p*-2,4(OSO_3−_)-(1→3)-α-L-Fuc*p*-4(OSO_3−_)-(1→3)-α-L-Fuc*p*-4(OSO_3−_)-(1→]_*n*_ (Alves, Mulloy, Moy, Vacquier and Mourão, 1998);**(G)** *Strongylocentrotus droebachiensis* [→4)-α-L-Fuc*p*-2(OSO_3−_)-(1→]_*n*_ (Vilela-Silva et al., 2002); **(H)** *Strongylocentrotus franciscanus* [3)-α-L-Fuc*p*-2(OSO_3−_)-(1→]_*n*_ (Vilela-Silva et al., 1999); **(I)** *L. varieagtus* II [3)-α-L-Fuc*p*-4(OSO_3−_)-(1→]_*n*_ (Cinelli et al., 2007); **(J)** *Echinometra lucunter* [→3-α-L-Gal*p*-2(OSO_3−_)-1→]_*n*_ (Alves et al., 1997); **(K)** *Glyptocidaris crenularis* [→3-β-L-Gal*p*-2(OSO_3−_)-1→3-β-L-Gal*p*-1→]_*n*_ (Castro et al., 2009); **(L)** *Herdmania monus* [→4)-α-L-Gal*p*-3(SO_3−_)-(1→]_*n*_ (Santos et al., 1992); **(M)** *Styela plicata* {→4)-α-L-Gal*p*-2[→1)- α-L-Gal*p*-3(OSO_3−_)]-3(OSO_3−_)-(1→}_*n*_ (Mourão and Perlin, 1987); **(N,O)** both *Botriocladia occidentalis* and *Gelidium crinale* express structures of [3-β-D-Gal*p*-1→4-α-Gal-1→]_*n*_ with different sulfation contents (Farias et al., 2000; Pereira et al., 2005b).

SFs are a class of SPs composed mostly of α-L-fucopyranosyl (Fuc*p*) units. SFs can be extracted mostly from marine organisms, such as sea-urchins (echinoidea), sea-cucumbers (holothuroidea) and brown algae (phaeophyta). These molecules are also known as fucoidans when isolated from brown algae. Besides Fuc*p*, the fucoidans are also composed of other sugars, such as xylose and uronic acids. This heterogeneous monosaccharide composition, associated with the lack or an unclear structural pattern of regularity and the presence of branching residues, makes the establishment of the structure-function relationships for algal molecules very hard (Pereira et al., 1999). On the other hand, the SFs isolated from invertebrates can reveal structures quite regular (Figures 1A–I). This type of structural pattern helps to achieve advanced structure-function correlations about their anticoagulant properties. Using the invertebrate SFs, we are able to understand which structural features are important for the anticoagulant activity of these molecules (Pereira et al., 1999; Pomin, 2009, 2012b,c). Below, some of these influential structural features on coagulation of the MSPs of well-defined chemical structures will be described.

SGs are a class of SPs composed of α-L-, α-D-, or β-D-galactopyranosyl (Gal*p*) units. SGs can be found in green algae (clorophyta), red algae (rodophyta), sea-urchins and ascidians which are also known as tunicates (ascidiacea). Like SFs, algal SGs have structures more complex than the regular and simpler invertebrate counterparts. In green algae, for example, the sulfation pattern is usually complex, with additional substitutions like pyruvates, and the possibility of branches (Farias et al., 2008). In red algae, although the SGs show heterogeneous sulfation patterns, they are usually composed of disaccharide repeating units of 3-linked β-D-Gal*p* and 4-linked α-Gal units in their backbones. Sometimes, the latter unit can be seen forming an extra carbon ring which results in an anhydro-sugar (Quinderé et al., 2014). This is an additional heterogeneity that enhances structural complexity in the red algal molecule. However, some red algal species can show very simple structures whose sulfation patterns vary accordingly to the species of extraction. Two examples of these structures are shown at panels N and O of Figure 1. Advanced structure-function correlations can be reached when these red algal SGs are used, as opposed to the more heterogeneous SGs from red or green algae. Conversely, the invertebrate SGs are very often composed of well-defined chemical structures (Figures 1J–M), which allow accurate structure-function correlations.

The information in the two previous paragraphs have made clear the advantages of the structures of the invertebrate SFs and SGs over the algal molecules, except few cases of red algal molecules (Figures 1N,O). These advantages come from the fact that the invertebrate molecules and the regular red algal SGs (Figure 1), which are composed of well-defined chemical structures, can be successfully used in structure-function relationship studies (Table 1). These studies allow prediction of the most influential structural combinations of the MSPs to achieve desirable anticoagulant responses. This knowledge is relevant to the development of these molecules as future therapeutic candidates.

**Table 1:** **Anticoagulant activities of MSPs of well-defined structures (Figure 1) measured by aPTT^a^ and by IC_50_ for thrombin citation(IIa) and factor Xa inhibition in the presence of antithrombin citation(AT) or heparin cofactor II (HCII)**.

**Polysaccharide**	**Source**	**Structure (Figure)**	**APTTcitation(IU/mg) ^a^**	**IC_50_ citation(μg/mL)**		
				**IIa/AT**	**IIa/HCII**	**Xa/AT**
3-linked sulfated α-L-fucans	*S. purpuratus* I	1E	76	0.3	0.3	2
	*S. purpuratus* II	1F	10	0.9	2	Nd^b^
	*S. pallidus*	1C	18	>500	>500	>500
	*L. variegatus* I	1B	3	>500	>500	>500
	*L. variegatus* II	1I	Nd	Nd	Nd	Nd
	*S. franciscanus*	1H	~2	>500	>500	250
	*L. grisea*	1A	<1	>500	>500	>500
4-linked sulfated α-L-fucans	*S. droebachiensis*	1G	<1	Nd	Nd	Nd
	*A. lixula*	1D	~2	150	150	>500
sulfated α-L-galactans	*E. lucunter*	1J	20	3	6	20
	*G. crenularis*	1K	Nd	Nd	Nd	Nd
	*H. monus*	1L	~2	>500	>500	>500
	*S. plicata*	1M	<1	>500	>500	>500
algal SGs	*B. occidentalis*	1N	93	0.02	1.1	2.5
	*G. crinale*	1O	65	0.02	25	1.5

Modified with permission from Pomin (2009).

a *The activity is expressed as international units/mg using a parallel standard curve based on the International Heparin Standard citation(193 units/mg)*.

b *Not determined*.

### Marine GAGs have unique structures

**Figure 2:**
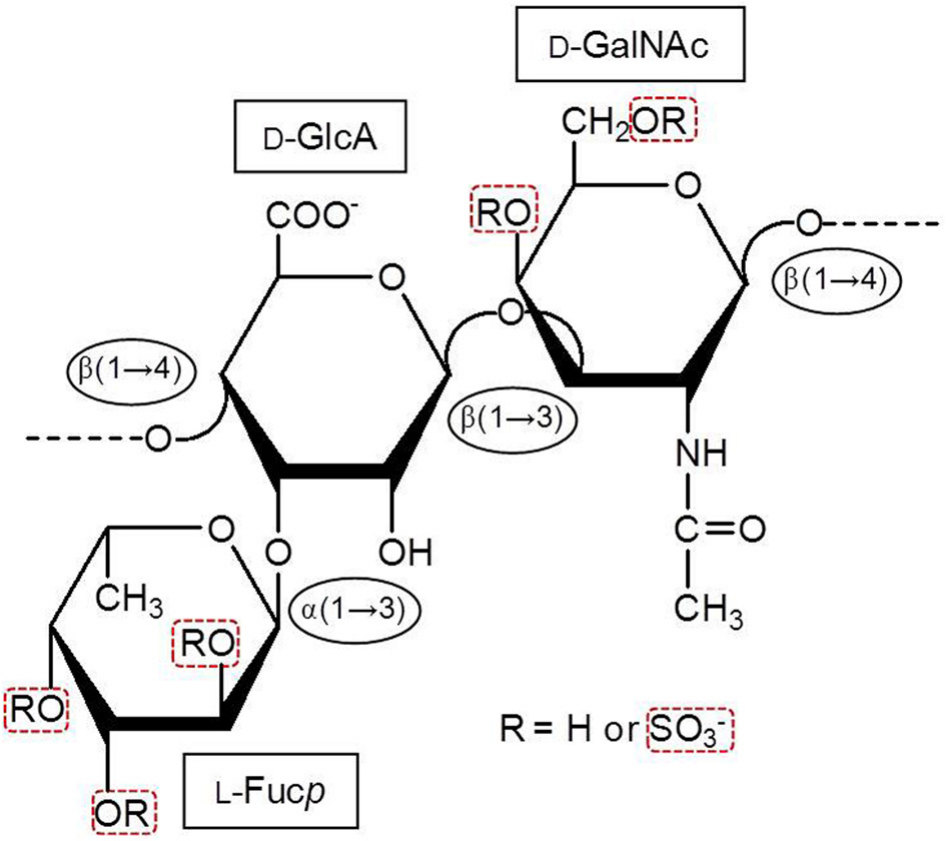
**Structural representation of the holothurian FucCS**. The monosaccharides are indicated by rectangles. They are α-l-fucopyranose (l-Fuc*p*), β-d-glucuronic acid (d-GlcA), and *N*-acetyl β-d-galactosamine (d-GalNAc). The glycosidic linkage types are indicated in ellipses. Modified with permission from Pomin (2014a).

Marine GAGs have different structures than those present in common mammal GAGs. For example, dermatan sulfate (DS) isolated from the ascidian species *Styela plicata* is composed of [→4)-α-L-IdoA-(2R^1^,3R^2^)-(1→3)-β-D-GalNAc-(4R^3^,6R^4^)-(1→]*_*n*_*, where IdoA is iduronic acid, GalNAc is *N*-acetyl galactosamine. The R^1^, R^2^, R^3^, and R^4^ are sulfate groups at 66, < 5, 94, and 6 percent, respectively (Pavão et al., 1998). The *S. plicata* is mostly 2-sulfated at the IdoA unit but largely 4-sulfated at the GalNAc unit. Conversely, the commonest mammalian DS is mostly composed of 2-sulfated IdoA units together with occasional C4 sulfation at GalNAc units. Another different GAG from marine invertebrates is the fucosylated chondroitin sulfate (FucCS) isolated from sea-cucumbers. These molecules are composed of the following structure {→3)-β-D-GalNAc-(1→4)-[α-L-Fuc*p*-(1→3)]-β-D-GlcA-(1→}_*n*_ (Figure 2) (Pomin, 2014a). The branching Fuc*p* unit can be sulfated at the 2, and/or 3 and/or 4-positions within different percentages according to the species of occurrence (Table 2). Conversely, the commonest chondroitin sulfates (CSs) of mammals are made of the following structure [→4)-β-D-GlcA-(1→3)-β-D-GalNAc-(1→]*_*n*_*, in which the GalNAc units can be either mostly 4-sulfated (CS-A) or predominantly 6-sulfated (CS-C) (Pomin et al., 2012).

**Table 2:** **Sulfation patterns (proportions of the branching sulfated fucose units) and the anticoagulant potential (measured by aPTT) of FucCS from 12 sea cucumber species analyzed so far**.

**Species**	**Fuc0S**	**Fuc3S**	**Fuc4S**	**Fuc2S4S**	**Fuc3S4S**	**aPTT**	**References**
*Ludwigothurea grisea*^a^	0	-	~49	~20	~17	55^b^	Mourão et al., 1996; Fonseca et al., 2009
*Pearsonothuria graeffei*	-	-	81.6	18.4	-	35^c^	Chen et al., 2011
*Holothuria vagabunda*	25.6	-	50.2	15.8	8.4	42^c^	Chen et al., 2011
*Stichopus tremulus*	-	-	24.8	22.4	52.8	135^c^	Chen et al., 2011
*Isostichopus badionotus*	-	-	4.1	95.9	-	183^c^	Chen et al., 2011
*Thelenata ananas*	0	~25	~22	~53	0	348^d^	Wu et al., 2010, 2012
*Stichopus japonicus*^e^	0	Nd^f^	11.1	55.6	33.3	Ns^g^	Yoshida and Minami, 1992
*Holothuria edulis*^h^	-	-	Nd	18	Nd	89^i^	Luo et al., 2013
*Apostichopus japonicas*^h^	-	-	Nd	45	Nd	116^i^	Luo et al., 2013
*Holothuria nobilis*^j^	-	Nd	Nd	-	Nd	59^i^	Luo et al., 2013
*Acaudina molpadioidea*^k^	-	-	-	-	-	Nc^l^	Ye et al., 2012
*Athyonidium chilensis*^k^	-	-	-	-	-	Nc^l^	Matsuhiro et al., 2012

*Reprint with permission from Pomin (2014a)*.

a *The CS backbone of FucCS from L. grisea has been extensively characterized. It is composed of GalNAc units with the following substitution percentages: 12% 4,6-di-sulfated, 53% 6-mono-sulfated, 4% 4-mono-sulfated, and 31% non-sulfated (Fonseca et al., 2009)*.

b *aPTT values expressed as international units/mg citation(IU/mg) using a parallel standard curve based on the International Heparin Standard (UFH) whose activity is 229 units/mg (Fonseca et al., 2009)*.

c *aPTT values expressed as international units/mg citation(IU/mg) using a parallel standard curve based on the International Heparin Standard citation(UFH) whose activity is 150 units/mg (Chen et al., 2011)*.

d *aPTT values expressed as international units/mg citation(IU/mg) using a parallel standard curve based on the International Heparin Standard citation(UFH) whose activity is 204 units/mg (Wu et al., 2012)*.

e *The CS backbone of this FucCS was mostly characterized as CS-E (Nagase et al., 1995), which is predominantly composed of 4,6-O-di-sulfated GalNAc units*.

f *Not determined*.

g *Not studied*.

h *Although the mono-4S and di-3S4S fucosyl units have been assigned in the FucCS of H. edulis and A. japonicas in Luo et al. (2013), the amounts of these units were not provided therein*.

i *aPTT values expressed as international units/mg citation(IU/mg) using a parallel standard curve based on the International Heparin Standard citation(UFH) whose activity is 212 units/mg (Luo et al., 2013)*.

j *The FucCS from H. nobilis was studied by NMR but the anomeric signals belonging to the fucose residues were rather to weak and broad to allow integration and further quantitation of their proportions. However, mono-3S, mono-4S, and di-3S4S fucosyl units were clearly observed (Luo et al., 2013)*.

k *Structures studied by Fourier transformed-infrared spectroscopy. Just a few structural features were raised. The sulfation patterns of FucCS from these two holothurian species are still an unknown*.

l *Not clear. Although the aPTT assay was undertaken and values were measured for different FucCSs concentration, the final values in IU/mg in comparison with a standard UFH curve were not provided*.

### Anticoagulant mechanisms of action of the MSPs

The effects of MSPs on hemostasis are the mostly studied medical activities of these compounds. The mechanisms of action in this particular clinical activity reside basically on the inhibition of some coagulation proteases like thrombin citation(IIa) and factor Xa, via their natural inhibitors, named serpins citation(serine-protease inhibitors). The most common serpins of this system are antithrombin citation(AT) and heparin cofactor II citation(HCII). Although at different degrees of response, the majority of the MSPs described in this review, the ascidian DS (Pavão et al., 1998; Vicente et al., 2004; Kozlowski et al., 2011), the sea-cucumber FucCS (Mourão et al., 1996; Mourão, 2004), the algal SFs and SGs (Pereira et al., 1999; Farias et al., 2000; Mourão, 2004) and the invertebrate SFs and SGs (Pereira et al., 1999, 2002; Pomin, 2012c) have either negligible or impressive effects in this serpin-dependent mechanism. The resultant anticoagulant activities of these MSPs (Tables 1, 2) are intimately and ultimately dependent on their structural features, as described below.

Besides the common serpin-dependent anticoagulant mechanism, the FucCS from the sea-cucumber *Ludwigothurea grisea*, which its fucosyl unit is mostly 2,4-di-sulfated (Figure 2; Table 2), and the SG from the red alga *Botryocladia occidentalis* (Figure 1N) have also shown serpin-independent anticoagulant mechanism (Glauser et al., 2008, 2009). Initially, their anticoagulant actions were primarily attributed by their capacity in potentiate the inhibition of factors Xa and IIa, via either AT or HCII. However, the sea-cucumber FucCS and the red algal SG are also known to inhibit the generation of factor Xa and IIa by interfering in the formation of the blood cofactor complexes at the surface of the cells. Factor Xa is activated mainly by the intrinsic tenase complex, while IIa is converted from II by the prothrombinase complex. FucCS and SG were shown the ability to inhibit the activation of these tenase and prothrombinase complexes (Glauser et al., 2008, 2009). The formation of these complexes is a key step for the generation and amplification of the coagulation cofactors. This serpin-independent mechanism has also been reported for other types of SGs (Quinderé et al., 2014). Unfortunately, advanced structure-function correlations are yet to be established for this novel mechanism. Therefore, the structural requirements necessary to achieve the anticoagulant activity via the serpin-independent mechanism are still unknown. For this reason, we will keep this new anticoagulant mechanism out of discussion here.

### The anticoagulant structural combinations

The anticoagulant structural features responsible for the anticoagulant activity of the MSPs can be determined from systematic analyses using the MSPs of well-defined chemical structures (Figure 1). On this way, comparing all the structures in Figure 1, one can discern structural similarities and differences in these molecules. For example, both the SF from *Strongylocentrotus franciscanus* (Figure 1H), and the SG from *Echinometra lucunter* (Figure 1J) present the same sulfation pattern (exclusive and entirely 2-sulfated), the same anomeric configuration (α-form), the same glycosidic linkage (1→3), and based on previous works (Pereira et al., 2005a), the same molecular mass (~100 kDa). Their single difference is the sugar type (Fuc*p* or Gal*p*). Interestingly, this single structural difference is itself enough to promote great changes in the anticoagulant outcomes of these homopolysaccharides. The 2-sulfated α-galactan from *E. lucunter* exhibits a significant anticoagulant activity monitored by the activated partial thromboplastin time (aPTT) method (Pomin, 2014b). The anticoagulant potential of this SG was determined to be 20 IU mg, although almost 10-fold less than unfractionated heparin (UFH) (Table 1). The specific anticoagulant assay with the purified proteases revealed that this SG enhances both IIa and factor Xa inhibition by either AT or HCII (Table 1). Conversely, the anticoagulant effect of 2-sulfated α-fucan from *S. franciscanus* is exclusively based on catalysis of AT inhibition over factor Xa, although it is 12.5-fold less active than the α-SG. This single effect on the Xa/AT system explains the much lower activity of the compound from *S. franciscanus* (aPTT of ~2 IU mg^−1^, 100-fold less active than UFH) since the anti-Xa activity has a relatively minor influence on the aPTT. This is an illustrative and typical example of a sugar-type-dependent biological effect of polysaccharides. The structural combination of 2-sulfation with 3-linked α-Gal*p* units results therefore in the anticoagulant response.

Based on this same systematic and comparative analysis, the SGs from the red algae *Botryocladia occidentalis* and *Gelidium crinale* were seen to exhibit identical backbones, and the same chain sizes (Farias et al., 2000; Pereira et al., 2005b). However, there are slight differences in their sulfation patterns (Figures 1M,O). As summarized in Table 1, these structural differences account for the 30% difference in anticoagulant activity as observed by the aPTT values of these algal macromolecules and the even greater difference in catalytic effect of the SP from *B. occidentalis* on the HCII-mediated anti-IIA activity, which was approximately 25-fold more than the catalytic effect of the SP from *G. crinale*. When the backbones of the red algal SGs are identical but bearing sulfation as their main difference, the anticoagulant activity seems to be proportionally dependent of the sulfation content (Table 1).

The structural requirements for the interaction of the MSP of well-defined chemical structures with the coagulation cofactors and their target proteases and inhibitors were determined to be very stereospecific (Mourão, 2004; Pomin and Mourão, 2008; Pomin, 2009). The site of sulfation has a major impact on the activity. This can be illustrated by the fact that 2,4-di-sulfated units have an amplifying effect on the AT-mediated anticoagulant activity in the series of 3-linked α-L-fucans (Figure 1; Table 1). Specific sulfation sites are required for the interaction with plasma serine-protease inhibitors. Note the occurrence of the 4-sulfated unit content in the 3-linked α-L-fucans vs. their anticoagulant activities (Table 1). *L. variegatus* (a single 4-sulfated unit/tetrasaccharide, Figure 1B, has 3 IU/mg of activity), *S*. *pallidus* (a double 4-sulfated unit/tetrasaccharide, Figure 1C, has 18 IU/mg of activity), and *S. purpuratus* isotype II (a double 4-sulfated unit/trisaccharide, Figure 1F, has 76 IU/mg of activity). This 4-sulfation is the structural motif required to enhance the inhibition of IIa by HCII. In contrast, the presence of 2-sulfated residues seems to have a deleterious effect on HCII-mediated anti-IIA activity of the polysaccharides (Mourão, 2004).

In the studies using the ascidian DS, Pavão and co-authors demonstrated that 4-sulfation at GalNAc units together with some 2-sulfation at the IdoA units is a structural motif of anticoagulant properties in these molecules (Pavão et al., 1998). This was observed based on the fact that DS from the species *Styela plicata* and *Halocynthia pyriformis* have large amounts of 4-*O*-sulfated GalNac units (94 and 99%, respectively), together with the reasonable amounts of 66 and 70% of 2-sulfation at the IdoA units. Their anticoagulant actions were measured by aPTT and shown to be 11 and 8 IU/mg, respectively; while the activity of the DS from bovine mucosa was shown to be only 2 units/mg (Pavão et al., 1998). Unlike the ascidian DSs, the mammalian DS is largely 2-sulfated at the IdoA unit and much less sulfated at the 4-position of the GalNAc units, as above-mentioned.

The sulfated fucosyl branches in the sea-cucumber FucCS were shown to be essential to their anticoagulant activities. This statement is based on the fact that when these branching units are removed, for example, by mild acid hydrolysis, or desulfated, by desulfation reactions, their anticoagulant effects disappears (Pomin, 2014a). Besides the necessary existence of these branching units, their sulfation patterns are also influential to the levels of anticoagulant activity. This can be seen from the structures and aPTT values of Table 2. The 2,4-di-sulfation in the fucosyl branching units appears to be the best sulfation pattern to the anticoagulant activity of this class of SPs. The importance of the 2,4-di-sulfation in Fuc*p* units was already pointed out in the work of Fonseca et al. (2009), as above for the AT-mediated anticoagulant activities of the series of 3-linked α-L-fucans (Figure 1, Table 1). In the work of Fonseca et al. (2009), the 2,4-di-sulfated Fuc*p* units were reported to be crucial to the anticoagulant activity of both sea-cucumber FucCS molecules and the linear SFs from invertebrates (Figure 1, Table 1).

## Major conclusions

Here, we have made clear the relevance of certain structural combinations of sulfation and glycosylation to the anticoagulant activity of the marine carbohydrates of well-defined chemical structures. For example, 2-sulfation together with 3-linked α-L-Gal*p* units, as found in the SG from the sea-urchin *E. lucunter* (Figure 1J), represents one of these structural combinations. Another combination is the 2,4-di-sulfation with α-L-Fuc*p* units. This combination can be found either in linear SFs of sea-urchins and sea-cucumbers (Figures 1A,B,F), or in the branched FucCS molecules (Figure 2, Table 2). The FucCS is a unique GAG found exclusively in sea-cucumbers. Another unique GAG from marine sources are the ascidian DS. For a good anticoagulant response of these GAGs, the 4-sulfation in GalNAc units combined with occasional 2-sulfation in IdoA units seems to be essential. When SGs from red algae exhibit simple backbones in which sulfation comprises the single modification and difference, sulfation content regardless the pattern seems to be relevant for the anticoagulant activity. In this case, the anticoagulant activity increases proportionally with the sulfation content. Finally, in invertebrate SFs, the 4-sulfation was observed to be a structural motif required to enhance the HCII-mediated inhibition of IIa. All these conclusions are summarized at Table 3 for a straightforward representation. Although we have clearly revised the relevance of sulfation and glycosylation to the anticoagulant activity of MSPs, molecular weights are also known to be influential to the biological activity (Melo et al., 2004; Quinderé et al., 2014). To ensure that our interpretation on the anticoagulant effects of MSPs have been based solely on sulfation and glycosylation patterns, our comparative analyses using the different MSP structures were performed on samples of similar molecular weights. This procedure disregards the contribution from differential molecular weights.

**Table 3:** **Summary of the structural requirements and effects in anticoagulation of the MSPs**.

**Structural requirement**	**Outcome**
2-sulfated 3-linked α-L-Gal*p*^a^	Enhance serpin (HCII and AT) inhibitory activity over the coagulation proteases (IIa and Xa)
2,4-disulfation in Fuc*p*^b^	
4-sulfated GalNAc + 2-sulfated IdoA in ascidian DS^c^	
Sulfation content in red algal homogeneous SGs^d^	
4-sulfation in invertebrate 3-linked SFs^e^	Enhance HCII-dependent IIa inhibition

a *(Pereira et al., 2005a)*.

b *(Fonseca et al., 2009)*.

c *(Pavão et al., 1998)*.

d *(Pereira et al., 2005b)*.

e *(Pereira et al., 2002)*.

One of the major goals of research programs involved with drug development nowadays, especially those related with potential carbohydrate-based drug candidates, is to understand the structural requirements of the new compounds in their specific therapeutic functions. Here, we have presented in a clear and straightforward way some of these structural requirements of the invertebrate and red algal SPs for their anticoagulant properties.

### Conflict of interest statement

The authors declare that the research was conducted in the absence of any commercial or financial relationships that could be construed as a potential conflict of interest.

## Abbreviations

aPTT: activated partial thromboplastin time
AT: antithrombin
DS: dermatan sulfate
Fuc*p*: L-fucopyranose
FucCS: fucosylated chondroitin sulfate
GAGs: glycosaminoglycans
GalNAc: N-acetyl D-galactosamine
Gal*p*: galactopyranose
GlcA: D-glucuronic acid
HCII: heparin cofactor II
IdoA: L-iduronic acid
MSPs: marine sulfated polysaccharides
SGs: sulfated galactans
SFs: sulfated fucans
SPs: sulfated polysaccharides
UFH: unfractionated heparin
IIa: thrombin
XIIa: factor XII activated.
